# Selective ß2-Adrenoceptor Agonists and Relevant Hyperlactatemia: Systematic Review and Meta-Analysis

**DOI:** 10.3390/jcm9010071

**Published:** 2019-12-27

**Authors:** Alina G. Liedtke, Sebastiano A. G. Lava, Gregorio P. Milani, Carlo Agostoni, Viola Gilardi, Mario G. Bianchetti, Giorgio Treglia, Pietro B. Faré

**Affiliations:** 1 Department of Internal Medicine, Ente Ospedaliero Cantonale, 6600 Locarno, Switzerland; Alina.Liedtke@eoc.ch (A.G.L.); PietroBenedetto.Fare@eoc.ch (P.B.F.); 2 Pediatric Cardiology Unit, Department of Pediatrics, Centre Hospitalier Universitaire Vaudois, and University of Lausanne, 1010 Lausanne, Switzerland; webmaster@sebastianolava.ch; 3 Pediatric Unit, Fondazione IRCCS Ca’ Granda Ospedale Maggiore Policlinico, 20122 Milan, Italy; carlo.agostoni@unimi.it; 4 Department of Clinical Sciences and Community Health, Università degli Studi di Milano, 20122 Milan, Italy; 5 Faculty of Biomedical Sciences, Università della Svizzera Italiana, 6900 Lugano, Switzerland; viola.gilardi9@gmail.com (V.G.); mario.bianchetti@usi.ch (M.G.B.); 6 Pediatric Institute of Southern Switzerland, Ospedale San Giovanni, 6500 Bellinzona, Switzerland; 7 Academic Education, Research and Innovation Area, General Directorate, Ente Ospedaliero Cantonale, 6500 Bellinzona, Switzerland; Giorgio.Treglia@eoc.ch; 8 Faculty of Biology and Medicine, University of Lausanne, 1000 Lausanne, Switzerland

**Keywords:** acidosis, Kussmaul breathing, lactate, lactic acid, ß_2_-adrenoceptor agonist

## Abstract

Selective ß_2_-agonists have been imputed as potential cause of l-hyperlactatemia since the 1970s. To document the prevalence of hyperlactatemia associated with selective ß_2_-agonists and to investigate the predisposing factors, we searched for published articles until April 2019 pertaining to the interplay of administration of selective ß_2_-agonists and circulating l-lactic acid in the Excerpta Medica, Web of Science, and the U.S. National Library of Medicine databases. Out of the 1834 initially retrieved records, 56 articles were included: 42 papers reporting individual cases, 2 observational studies, and 12 clinical trials. Forty-seven individual patients receiving a selective ß_2_-agonist were found to have l-lactatemia ≥5.0 mmol/L, which decreased by ≥3.0 mmol/L or to ≤2.5 mmol/L after discontinuing (N = 24), reducing (N = 17) or without modifying the dosage of the selective ß_2_-agonist (N = 6). Clinical trials found that l-lactic acid significantly increased in healthy volunteers administered a ß_2_-agonist. l-lactatemia ≥5.0 mmol/L was observed in 103 (24%) out of 426 patients with asthma or preterm labor managed with a selective ß_2_-agonist and was more common in patients with asthma (30%) than in premature labor (5.9%). A significant relationship was also noted between l-lactate level and intravenous albuterol dose or its circulating level. In conclusion, relevant l-hyperlactatemia is common on high dose treatment with a selective ß_2_-agonist.

## 1. Introduction

Excess (≥2.5 mmol/L) blood lactic acid level and, less frequently, lactic acidosis, may result from poor tissue oxygen delivery, from inherited or acquired metabolic defects and from drugs such as biguanides, some antiretrovirals, the antibiotic linezolid, and the hypnotic sedative propofol [1,2]. Selective ß_2_-adrenoceptor agonists are worldwide prescribed to manage bronchial obstruction, to prevent premature delivery and, less frequently, to treat hyperkalemia. ß_2_-adrenoceptor activation increases lactic acid synthesis in skeletal muscle cells [3]. Unsurprisingly, therefore, selective ß_2_-adrenoceptor agonists have been imputed as a cause of hyperlactatemia since the second half of the 70s [4].

Available guidelines do not mention the association of management with selective ß_2_-adrenoceptor agonists and hyperlactatemia [5,6,7]. The aim of this study was to document the prevalence and predisposing factors for hyperlactatemia associated with selective ß_2_-agonists.

## 2. Methods

### 2.1. Literature Search Strategy

A systematic search of scientific articles investigating the prevalence and predisposing factors for hyperlactatemia associated with selective ß_2_-agonists was performed by using the Excerpta Medica, Web of Science, and the U.S. National Library of Medicine databases (PROSPERO REGISTRATION NUMBER: CRD42019139789). The literature search was conducted until April 2019. The search algorithm used was a combination of different key words and Medical Subject Heading terms: (hyperlactatemia OR lactate OR lactic acid OR lactic acidosis OR metabolic acidosis) AND (albuterol OR bitolterol OR carmoterol OR fenoterol OR formoterol OR indacaterol OR metaproterenol OR procaterol OR rimiterol OR ritodrine OR salbutamol OR salmeterol OR terbutaline OR ß_2_-adrenoceptor agonist OR beta-2-agonist). Reports published in Dutch, English, French, German, Italian, Portuguese, or Spanish after 1970 as full-length articles or letters on the topic of interest were considered. We also scanned the references of all included articles for additional reports. We employed the principles underlying the U.K. Economic and Social Research Council guidance on the conduct of narrative synthesis and the “Preferred Reporting Items for Systematic reviews and Meta-Analyses” (PRISMA) statement.

### 2.2. Selection Criteria and Data Extraction

We included reports detailing individual subjects with clinically relevant hyperlactatemia (≥5.0 mmol/L) after taking either an intravenous or a nebulized selective ß_2_-agonist and a decrease in lactate level by ≥3.0 mmol/L or to a level of ≤2.5 mmol/L after discontinuing the ß_2_-agonist or reducing its dosage. Individual cases of relevant hyperlactatemia that improved without discontinuing or reducing the selective ß_2_-agonist were also included. Observational studies and clinical trials addressing the prevalence of relevant hyperlactatemia or the interplay between the metabolism of ß_2_-agonists and that of lactic acid were also retained. Cases managed with biguanides, antiretrovirals, linezolid, or propofol, or with inherited enzyme defects responsible for excess of this acid were excluded.

From each included report, information on demographics, drug name and route of administration of the selective ß_2_-agonist, co-medication with corticosteroids, ipratropium, theophylline, and underlying clinical condition was also collected. If needed, authors of original articles were contacted to provide missing data or verify the accurateness of reported information.

Literature selection and data extraction were performed independently by two investigators. When results were incongruent, conflicts were resolved by reaching a consensus and, if the discrepancy stood, a third researcher was consulted.

### 2.3. Study Quality Assessment

The Grading of Recommendations Assessment, Development and Evaluation (GRADE) score, which may be very low, low, moderate, or high, was used to appraise the quality of the observational and clinical trials included in this review.

## 3. Analysis

Continuous data are presented as median and interquartile range or as ‘box and whisker plot’, dichotomous data as relative frequency and confidence interval. The Cohen index was used to assess the agreement between investigators on the application of the inclusion and exclusion criteria, the Kruskal–Wallis test to compare continuous variables and the Fisher test to compare dichotomous variables.

We performed a pooled analysis about the prevalence of significant hyperlactatemia in patients receiving a selective ß_2_-agonist using data retrieved from the selected observational studies and clinical trials. A random-effects model was used for statistical pooling of the data, taking into account the heterogeneity among studies. The different weight of each study in the pooled analysis was related to the different sample size. Pooled data were presented with their respective 95% confidence interval (95% CI) values, and data were displayed using plots. Heterogeneity was estimated by using the I^2^ index, which describes the percentage of variation across studies that is due to heterogeneity rather than chance. Publication bias was assessed through the Egger’s test. Statistical analyses were performed using the StatsDirect software version 3 (StatsDirect Ltd., Cambridge, UK) and the Meta-analyse Software (Brown University, Providence, RI, USA).

Anticipating the possible occurrence of a significant heterogeneity (I^2^ index > 50%), subgroup analyses based on the type of studied population (a. healthy subjects, b. adults with asthma, c. children with asthma, and d. females with premature labor) or route of administration (a. intravenous or subcutaneous, b. nebulized, c. both intravenous and nebulized) were planned. Statistical significance was assigned at *p* < 0.05.

## 4. Results

### 4.1. Literature Search Results

**Figure 1:**
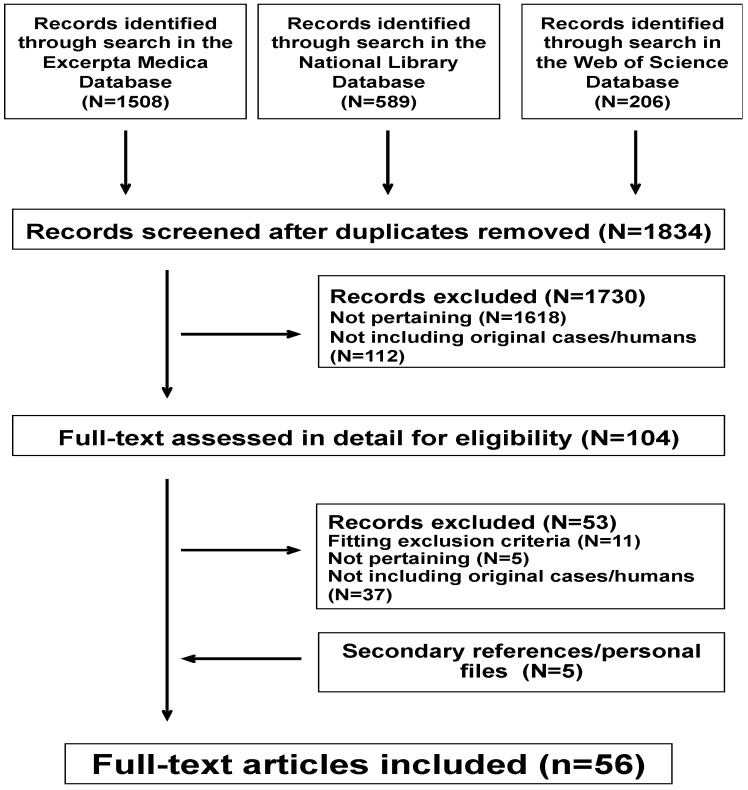
Flowchart of the literature search process.

The literature search process is recapitulated in Figure 1. The agreement between the two investigators on the application of the exclusion and inclusion criteria was 0.88. For the final analysis, we retained 56 reports [4,8,9,10,11,12,13,14,15,16,17,18,19,20,21,22,23,24,25,26,27,28,29,30,31,32,33,34,35,36,37,38,39,40,41,42,43,44,45,46,47,48,49,50,51,52,53,54,55,56,57,58,59,60,61,62]: 42 reporting individual cases [4,8,9,10,11,12,13,14,15,16,17,18,19,20,21,22,23,24,25,26,27,28,29,30,31,32,33,34,35,36,37,38,39,40,41,42,43,44,45,46,47,48], two observational studies [60,61] and 12 clinical trials [49,50,51,52,53,54,55,56,57,58,59,62]. The 56 reports were published between 1978 and 2019, in English (N = 46), French (N = 6), Spanish (N = 3) and Italian (N = 1).

### 4.2. Individual Cases

**Figure 2:**
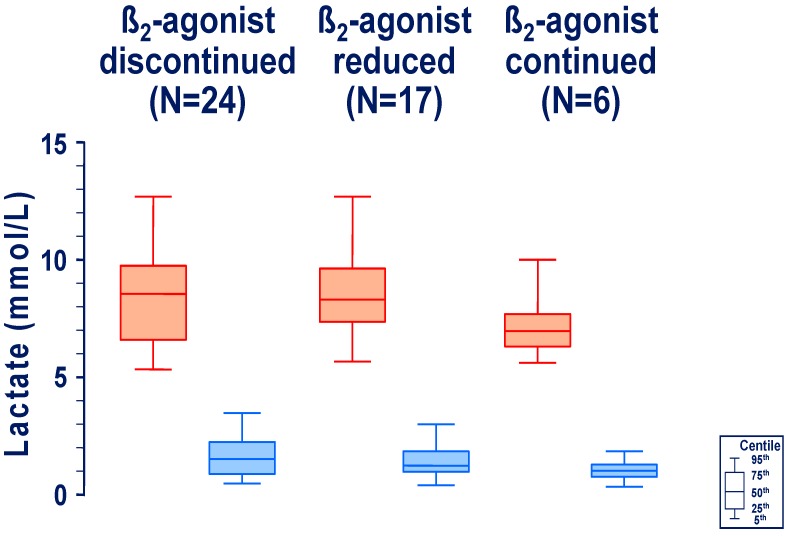
l-lactic acid level in 47 individual cases with relevant l-hyperlactatemia (≥5.0 mmol/L) after taking either an intravenous or a nebulized ß_2_-agonist (red color; ●) and with a decrease (blue color, ●) in l-lactate level by ≥3.0 mmol/L or to ≤2.5 mmol/L, 3 to 56, median 13 h after discontinuing, reducing or without modifying the dosage of the ß_2_-agonist. The results are given as ‘box and whisker plot’: bottom and top of box 25th and 75th centile, respectively, middle of box 50th centile (the median), ends of whiskers 5th and 95th centile, respectively.

Forty-two articles [4,8,9,10,11,12,13,14,15,16,17,18,19,20,21,22,23,24,25,26,27,28,29,30,31,32,33,34,35,36,37,38,39,40,41,42,43,44,45,46,47,48] reported on 47 patients (Table 1), who were found to have a blood lactic acid level ≥5.0 mmol/L on treatment with a short-acting (N = 46) or a long-acting (N = 1) ß_2_-agonist, which subsequently decreased by ≥3.0 mmol/L or to ≤2.5 mmol/L (Figure 2). This was noticed 3 to 56, median 13 h later: in 24 cases after discontinuing [4,9,10,11,14,15,16,18,19,22,24,25,29,32,33,34,36,38,40,41,47,48], in 17 cases after reducing [12,13,20,22,23,26,28,30,31,35,37,39,42,43,45], and in 6 cases (all of these patients were affected by asthma) without modifying [8,12,17,21,27,46], the dosage of the selective ß_2_-agonist. None of the patients was managed with further drugs potentially associated with hyperlactatemia.

### 4.3. Observational Studies and Clinical Trials

**Figure 3:**
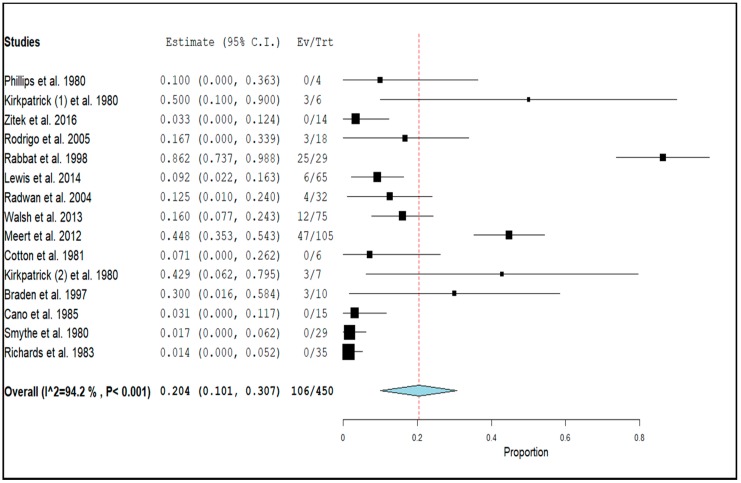
Forest plot of observational studies and clinical trials and pooled prevalence (dotted line) of relevant hyperlactatemia (≥5.0 mmol/L) associated with ß_2_-agonists, including 95% confidence intervals (95% CI). The size of the squares is related to the weight of each study. The horizontal lines indicate the 95% CI values for each study, whereas the horizontal diameter of the rhombus indicates the 95% CI value for the pooled prevalence. One clinical trial reported the results both on healthy volunteers (Kirkpatrick (1) et al., 1980) and on females with premature labor (Kirkpatrick (2) et al., 1980).

The interplay between the metabolism of a selective ß_2_-agonist and that of lactic acid was investigated in two observational studies [60,61], six uncontrolled clinical trials [49,52,53,57,58,59], three non-randomized controlled trials [51,54,55], and three randomized controlled trials [50,56,62]. The GRADE score was very low in one [61], low in five [42,52,58,59,60], moderate in five [51,53,54,55,57], and high in three [50,56,62] articles. The mentioned studies included a total of 450 subjects treated with a selective ß_2_-agonist. The pooled prevalence of relevant hyperlactatemia in subjects receiving ß_2_-agonists was 20.4% (95% CI: 10.1–30.7), as shown in Figure 3.

**Figure 4:**
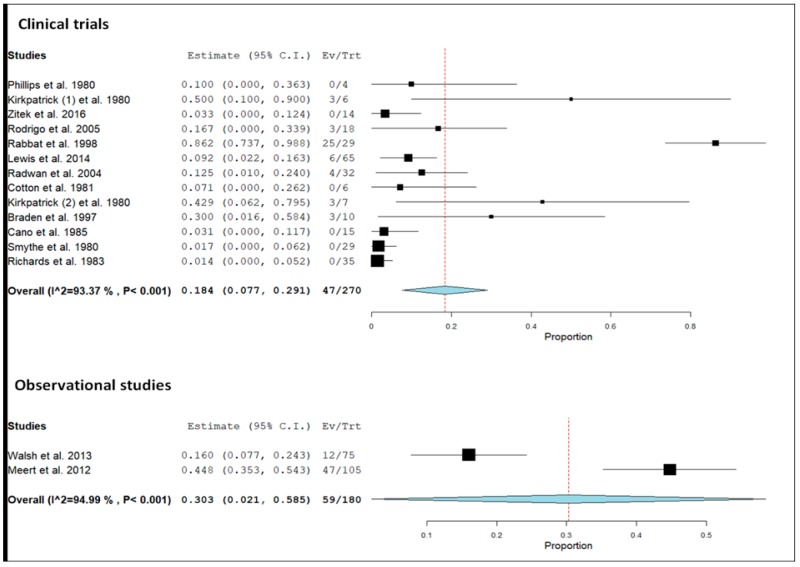
Forest plot of the 12 clinical trials (upper panel) and the two observational studies (lower panel) and pooled prevalence (dotted line) of relevant hyperlactatemia (≥5.0 mmol/L) associated with ß_2_-agonists in the two types of study design, including 95% confidence intervals (95% CI). The size of the squares is related to the weight of each study. The horizontal lines indicate the 95% CI values for each study, whereas the horizontal diameter of the rhombus indicates the 95% CI value for the pooled prevalence. One clinical trial reported the results both on healthy volunteers (Kirkpatrick (1) et al., 1980) and on females with premature labor (Kirkpatrick (2) et al., 1980).

A substantial heterogeneity among studies (I^2^ index = 94.2%) was detected. A similar heterogeneity was also observed when clinical trials and observational studies were separately considered (Figure 4).

**Figure 5:**
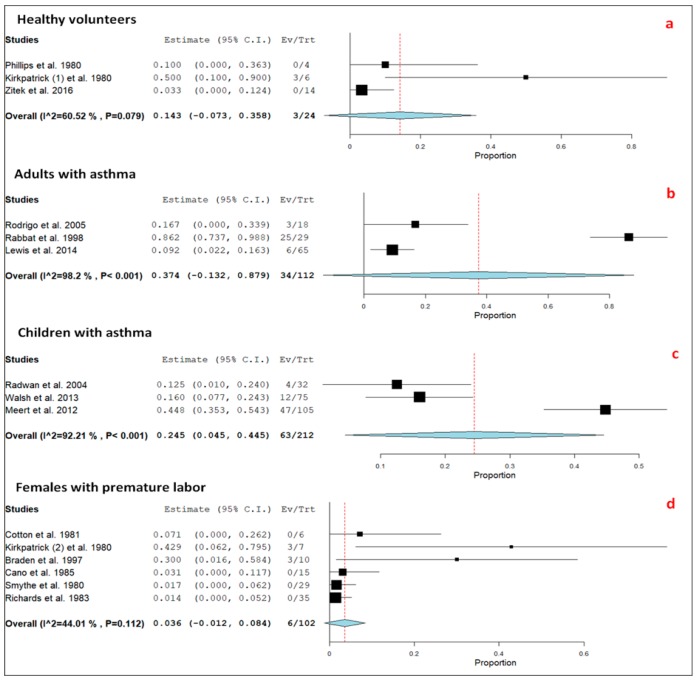
Forest plots of pooled prevalence including 95% confidence interval (95% CI) values of relevant hyperlactatemia (≥5.0 mmol/L) associated with selective ß_2_-agonists, in the different study populations: (**a**) healthy volunteers; (**b**) adults with asthma; (**c**) children with asthma; and (**d**) females with premature labor. The size of the squares is related to the weight of each study. The horizontal lines indicate the 95% CI values for each study, whereas the horizontal diameter of the rhombus indicates the 95% CI value for the pooled prevalence.

Among the different types of study populations, circulating lactic acid significantly increased in healthy volunteers [49,50] administered intravenous (N = 4; by about 2.0 mmol/L) or nebulized (N = 14; by about 0.8 mmol/L) albuterol. A significant increase in circulating lactate was also observed in 6 healthy volunteers after intravenous ritodrine [51]. In this study population, hyperlactatemia was relevant (≥5.0 mmol/L) only in a small, not significant minority of cases (N = 3; 13%). A total of 426 patients (214 adults and 212 children) with acute asthma (N = 324) or preterm labor (N = 102) managed with nebulized, intravenous, subcutaneous, or both nebulized and intravenous albuterol (N = 324), ritodrine (N = 62) or terbutaline (N = 40) were investigated in the 12 clinical trials. Relevant hyperlactatemia was observed in 103 (24%) cases and was significantly (*p* < 0.0001) more common in asthma patients (N = 97; 30%) than in females with premature labor (N = 6; 5.9%). Among the 324 aforementioned patients affected by acute asthma, the prevalence of relevant hyperlactatemia was identical in patients ≤18 years of age (63 out of 212, 30%) and in older subjects (34 out of 112, 30%). In 42 pediatric subjects managed with intravenous albuterol, a significant (*p* < 0.02) correlation was observed between intravenous albuterol dose and lactate level [61]. In addition, in 65 adult patients, a significant (*p* < 0.0001) positive correlation was noted between circulating albuterol and lactate level [62]. Finally, Radwan et al. found significantly higher lactic acid levels in children with more severe asthma attacks [58]. The subgroup analysis of the pooled prevalence of relevant hyperlactatemia taking into account the different studied population disclosed an I^2^ index of 44% in the group of women with premature labor (Figure 5). This index was >50% in the remaining three subgroups.

**Figure 6:**
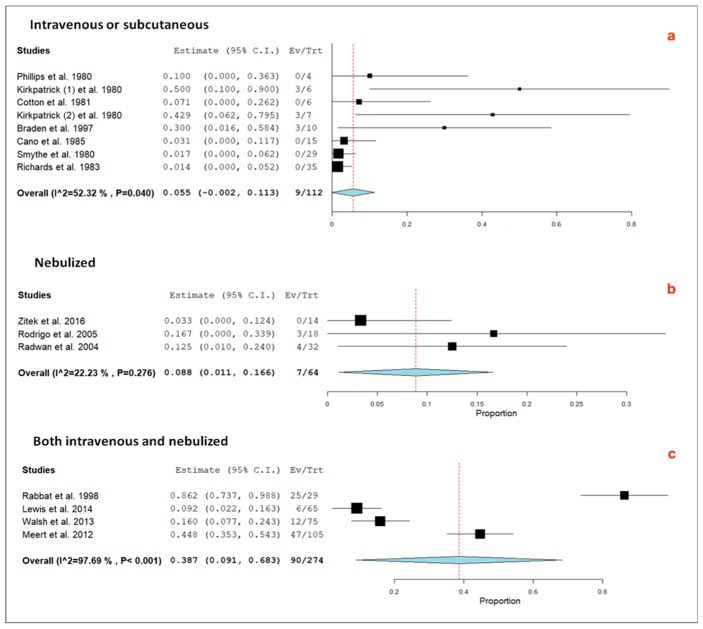
Forest plots of pooled prevalence including 95% confidence interval (95% CI) values of relevant hyperlactatemia (≥5.0 mmol/L) associated with selective ß_2_-agonists, according to the various routes of drug administration: (**a**) intravenous or subcutaneous; (**b**) nebulized; (**c**) both intravenous and nebulized. The size of the squares is related to the weight of each study. The horizontal lines indicate the 95% CI values for each study, whereas the horizontal diameter of the rhombus indicates the 95% CI value for the pooled prevalence. The Egger’s test did not detect a significant publication bias (*p* > 0.05).

A total of seven studies included subjects managed exclusively with intravenous or subcutaneous ß2-agonists (N = 112), three studies included subjects managed exclusively with nebulized ß_2_-agonists (N = 64), and four studies included subjects managed both with intravenous and nebulized ß_2_-agonists (N = 274). The subgroup analysis taking into account the route of administration disclosed an I^2^ index of 22% in the group of subjects managed with nebulized ß_2_-agonists and >50% in the remaining two groups (Figure 6).

## 5. Discussion

The results of the first systematic review and meta-analysis on selective ß_2_-agonist associated hyperlactatemia can be summarized as follows. First, administration of either an intravenous or a nebulized ß_2_-agonist may be followed by an increased lactic acid level in healthy volunteers, in both pediatric and adult patients with an acute bronchial obstruction or hyperkalemia, in women with preterm labor, and following voluntary intoxication. The effect on lactic acid of albuterol, the most frequently prescribed ß_2_-agonist, is dose-dependent and correlates with its blood level. Second, management with a high dose of a short-acting ß_2_-agonist is associated with clinically relevant hyperlactatemia (≥5.0 mmol/L) in every third patient with asthma admitted to an intermediate or intensive care unit. Third, hyperlactatemia has been found to resolve after stopping, reducing, or even continuing the ß_2_-agonist.

Acute asthma and chronic obstructive pulmonary disease, the most common indications for management with selective ß_2_-agonists, may per se be associated with hyperlactatemia. Poor tissue oxygen delivery might contribute to hyperlactatemia in this setting [63,64]. However, a severely impaired oxygen delivery is required to cause hyperlactatemia [63,64]. As a consequence, it is currently assumed that hyperlactatemia predominantly results from lactic acid overproduction by respiratory muscles performing increased work and, to a lesser extent, from its reduced elimination caused by liver hypoperfusion [63,64]. In vitro and in vivo studies have elucidated the mechanism by which ß_2_-agonists cause lactic acid generation: ß_2_-adrenoceptor activation stimulates production of lactic acid in skeletal muscle (but not in other tissues) through exaggerated aerobic glycolysis [3,65].

In the past, an increased ratio of lactate to pyruvate concentration in blood has been deemed to distinguish hyperlactatemia due to poor tissue oxygen delivery from that without [66]. Since recent observations demonstrate that this ratio may be an inaccurate marker of tissue oxygen delivery, this information was not addressed in this review [66].

In asthma exacerbation managed with high dose selective ß_2_-agonists, tachypnea represents a diagnostic challenge. Given the high prevalence of relevant hyperlactatemia on treatment with these drugs, tachypnea may result from airway obstruction, Kussmaul breathing caused by lactic acidosis, or both. In this setting, serial physical examination (and perhaps, in older children and adults, peak flow measurement) are advised to appreciate airway obstruction. Many currently available point-of-care blood gas analyzers also assess lactic acid. In our opinion, however, the determination of this acid in severe asthma managed with a ß_2_-agonist is likely to be currently underappreciated.

Our analysis complements the results of a very recent review which included patients ≥13 years of age with hyperlactatemia associated with the administration of both selective and non-selective ß_2_-agonists [2].

This report has at least three limitations. First, the main limitation of our study was the detected heterogeneity. The subgroup analyses taking into account the route of drug administration showed a reduced statistical heterogeneity among the studies. Therefore, this finding points out that the different route of drug administration is a significant cause of heterogeneity among the included studies. In addition, the heterogeneity observed in healthy subjects and in patients with asthma is likely due to the variable doses of selective ß_2_-agonists. Furthermore, in asthma patients, excess lactic acid level may occur as a consequence of increased respiratory muscle work (even without treatment with a ß_2_-agonist). Regrettably, all but one report did not assess asthma exacerbation severity, making it impossible to correlate circulating lactic acid level with disease severity. Second, available data can sketch out the management of hyperlactatemia only in very broad terms. Third, the literature does not permit to identify the role of co-medication with corticosteroids, ipratropium, or theophylline in the occurrence of ß_2_-agonist-associated hyperlactatemia.

## 6. Conclusions

The potential of selective ß_2_-agonists to produce hypocalcemia, hypomagnesemia, hypophosphatemia, and especially hypokalemia and hyperglycemia is well recognized but often overlooked in clinical practice [2,67,68,69]. The present review of the literature points out that relevant hyperlactatemia is also common (about 20%) on high dose treatment with these agents. In patients with acute asthma, healthcare providers might misinterpret relevant hyperlactatemia as worsening of respiratory disease.

## Figures and Tables

**Table 1:** Patient demographics and characteristics of the 47 individual cases 2.0 to 66, median 26 years of age with relevant hyperlactatemia (≥5.0 mmol/L) after taking a selective ß_2_-agonist. Data are presented as frequency or as median and interquartile range.

**Demographics**	
Gender (female:male), N	32:15
Age	
years	26 [16,17,18,19,20,21,22,23,24,25,26,27,28,29,30,31,32,33,34,35,36,37,38,39,40,41,42,43,44]
<18 years:≥18 years, N	16:31
**Underlying Conditions**	
Acute asthma, N	41
Intraoperative bronchospasm, N	3
Hyperkalemia, N	1
Premature labor, N	1
Voluntary intoxication, N	1
**Agent**	
Albuterol, N	35
Terbutaline, N	2
Albuterol and terbutaline, N	7
Metaproterenol, N	1
Ritodrine, N	1
Salmeterol, N	1
**Route of Administration**	
Nebulized, N	37
Intravenous, N	3
Nebulized and intravenous, N	7
**Further Medication**	
Corticosteroids, N	41 *
Ipratropium, N	25
Theophylline, N	14

* systemic administration in 40 cases.
